# Radiomics-based machine learning models to distinguish between metastatic and healthy bone using lesion-center-based geometric regions of interest

**DOI:** 10.1038/s41598-022-13379-8

**Published:** 2022-06-14

**Authors:** Hossein Naseri, Sonia Skamene, Marwan Tolba, Mame Daro Faye, Paul Ramia, Julia Khriguian, Haley Patrick, Aixa X. Andrade Hernandez, Marc David, John Kildea

**Affiliations:** 1 Medical Physics Unit, McGill University, Montreal, QC Canada; 2 Department of Radiation Oncology, McGill University Health Center (MUHC), Montreal, QC Canada; 3 University of Texas Southwestern Medical Center, Dallas, TX USA

**Keywords:** Bone metastases, Machine learning, Image processing, Predictive medicine, Software

## Abstract

Radiomics-based machine learning classifiers have shown potential for detecting bone metastases (BM) and for evaluating BM response to radiotherapy (RT). However, current radiomics models require large datasets of images with expert-segmented 3D regions of interest (ROIs). Full ROI segmentation is time consuming and oncologists often outline just RT treatment fields in clinical practice. This presents a challenge for real-world radiomics research. As such, a method that simplifies BM identification but does not compromise the power of radiomics is needed. The objective of this study was to investigate the feasibility of radiomics models for BM detection using lesion-center-based geometric ROIs. The planning-CT images of 170 patients with non-metastatic lung cancer and 189 patients with spinal BM were used. The point locations of 631 BM and 674 healthy bone (HB) regions were identified by experts. ROIs with various geometric shapes were centered and automatically delineated on the identified locations, and 107 radiomics features were extracted. Various feature selection methods and machine learning classifiers were evaluated. Our point-based radiomics pipeline was successful in differentiating BM from HB. Lesion-center-based segmentation approach greatly simplifies the process of preparing images for use in radiomics studies and avoids the bottleneck of full ROI segmentation.

## Introduction

In recent years, radiomics-based machine learning (ML) classifiers have shown great potential for use in the early detection of bone metastases (BM) and in assessing response of BM to radiotherapy (RT)^1–20^. However, in order to be clinically acceptable, radiomics models must be trained on large data sets of real-world images. This is challenging as the full 3D segmentation of BM on planning-CT images is time-consuming for radiation oncologists in the clinical context. Often, in the interest of time and given the low doses used in palliative RT, radiation oncologists only delineate treatment field boundaries when treating BM, and they do not fully contour individual BM lesions. As a result, most of the published BM radiomics studies to date were trained and tested with relatively small sample sizes (see Table 1), which diminishes their generalizability and their applicability to clinical RT planning. Motivated by the need for large real-world BM data sets, the objective of this study was to determine if a radiomics model can be trained to distinguish BM from healthy bone (HB) using BM lesions denoted as points on planning-CT images rather than using full 3D segmentation.

### Radiomics for metastases detection

Radiomics is an automated feature generation method for the extraction of hundreds of quantitative phenotype (radiomics features) from radiology images^21,22^. ML algorithms can be trained to find relationships between radiomics features and cancer outcomes if provided with sufficient and appropriate data. There are three main steps in the training phase of a typical radiomics study. These include: (1) manual or semi-automated segmentation of regions of interest (ROIs) on patients’ images, (2) feature extraction from the segmented ROIs, and (3) generation of a statistical or ML model to correlate extracted features to each patient’s endpoint data such as their cancer outcome or other clinically-measured biomarkers^8^.

In addition to the need for adequate sample sizes, which is the main motivation behind this study, a radiomics model must overcome two important challenges in order to be reliable in a clinical context. First, it must be clinically reproducible. This is challenging because different radiomics studies use different subsets of radiomics features to achieve optimal models. The variations in published feature selection approaches make radiomics models less clinically reproducible^23,24^. Therefore, to achieve a clinically-reliable radiomics model, it is important to study and account for the effect of the variation in feature selection (FS) methods^25–27^.

Depending on the endpoint of interest, various ML classifiers may be used in a radiomics model. Support vector machine (SVM), Bayesian network (BN), multivariate logistic regression (MLR), k-nearest neighbor (kNN), decision trees (DT), random forests (RF), neural network (NNet), and convolutional neural networks (CNN) are among the ML classifiers that are most commonly used in radiomics-based ML models^8–20^. The feasibility of using radiomics-based ML models to distinguish between benign and malignant bone lesions has been reported in previous studies^1–4,6,7^. The main details of these studies are summarized in Table 1.

**Table 1:** Radiomics-based ML models reported in the literature for distinguishing bone lesions.

Author year	Sample size$^{*}$	Imaging Modality	ROI	Labels	Classifier	Performances (AUC, A, P, R)$^\dag$
Perk et al.^1^	36	PET/CT	Manual	Benign and metastatic	RF	0.95, 0.88, 0.88, 0.89
Suhas and Mishra^2^	74	Diagnostic-CT	Semi-automated$^\ddag$	Benign and malignant	RF	0.90, 0.92, 0.92, 0.91
Acar et al.^3^	75	PET/CT	Manual	Responded and metastatic	kNN	0.76, 0.74, 0.74, 0.74
Suhas and Kumar^4^	100	Diagnostic-CT	Semi-automated$^\ddag$	Benign and malignant	SVM	0.86, -, 0.85, 0.88
Homayounieh et al.^5^	103	Dual-Energy CT	Semi-automated$^\ddag$	Benign and malignant	RF	0.79, 0.78, 0.72, 0.79
Hong et al.^6^	177	CT	Manual	Bone island and metastases	RF	0.96, 0.80, 0.96, 0.86
Sun et al.^7^	206	Diagnostic-CT	Manual	Benign and malignant	MLR	0.82, 0.86, 0.93, 0.77

$^{*}$Sample size is the total number of samples. $^{\dag }$*A* accuracy, *P* precision (Specificity), *R* recall (sensitivity). $\ddag$In the semi-automated segmentation methods an expert was required to check and modify the computer-segmented ROIs slice-by-slice.

The radiomics-based ML models listed in Table 1 are not readily applicable to our clinical context, palliative RT for BM, for three reasons. First, they have relatively small sample sizes, an inherent problem for generalizability. Second, they require full 3D lesion segmentation, which is challenging to achieve clinically when planning palliative RT for BM. Finally, they were trained on images acquired using diagnostic-CTs or hybrid imaging modalities, whereas palliative RT planning is mostly done on planning-CT (simulation-CT) images alone.

With the above limitations in mind, in this study, we investigated the feasibility of developing a fast and reliable radiomics-based ML pipeline capable of differentiating between BM and HB in RT planning-CT images of cancer patients using just geometric ROIs centered on expert-identified lesion point locations. We investigated the effect of using ROIs with different sizes and geometric shapes. We also examined the performance of different FS methods and ML classifiers in achieving the optimal BM detection pipeline.

## Materials and methods

### Patient selection

**Figure 1:**
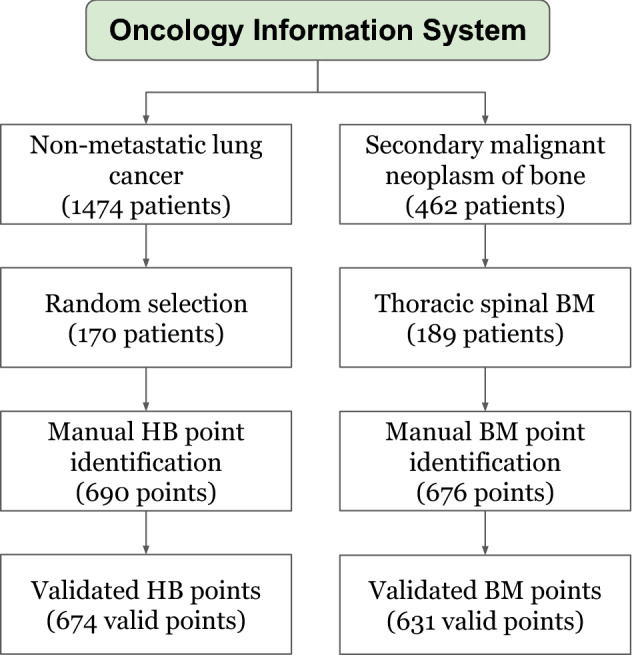
Flow chart of patient selection.

The planning-CT images of BM and HB patients used in this study were collected from the Oncology Information System at our institution. Our patient selection procedure is presented in Fig. 1.

BM samples were from patients who received palliative RT for a secondary malignant neoplasm of bone in the thoracic spine between January 2016 and September 2019. HB samples were from individuals who received curative RT for non-metastatic lung cancer (as their CT images covered the same anatomy) during this period.

In total, we found 189 BM patients (96 male and 93 female; mean age± standard deviation (SD), 69 ± 13 years) and 1474 HB patients in our database. To reduce the large imbalance between the number of BM and HB patients, we randomly shuffled the HB sample (in a Microsoft Excel file) and selected the first 170 patients (86 male and 84 female; mean age 71 ± 12 years) to include in our study (see Fig. 1).

### Planning-CT images

All planning-CT images were generated using one of three Philips’ Brilliance Big Bore RT CT scanners at our institution with the acquisition parameters provided in Table 2. Planning-CT DICOM files were manually de-identified and exported to a secured hard drive from the Eclipse radiation therapy treatment planning software (Varian Medical Systems, Palo Alto, California), into which they had been previously imported for RT planning.

**Table 2:** Planning-CT image acquisition parameters.

Tube voltage (kV)	Tube current (mA)	Exposure (mm)	Field of view (pixel)	Matrix size (mm)	Slice thickness (mm)	Pixel spacing
120	165–366	240–450	600	512 $\times$ 512	3.0	0.77–1.37

**Figure 2:**
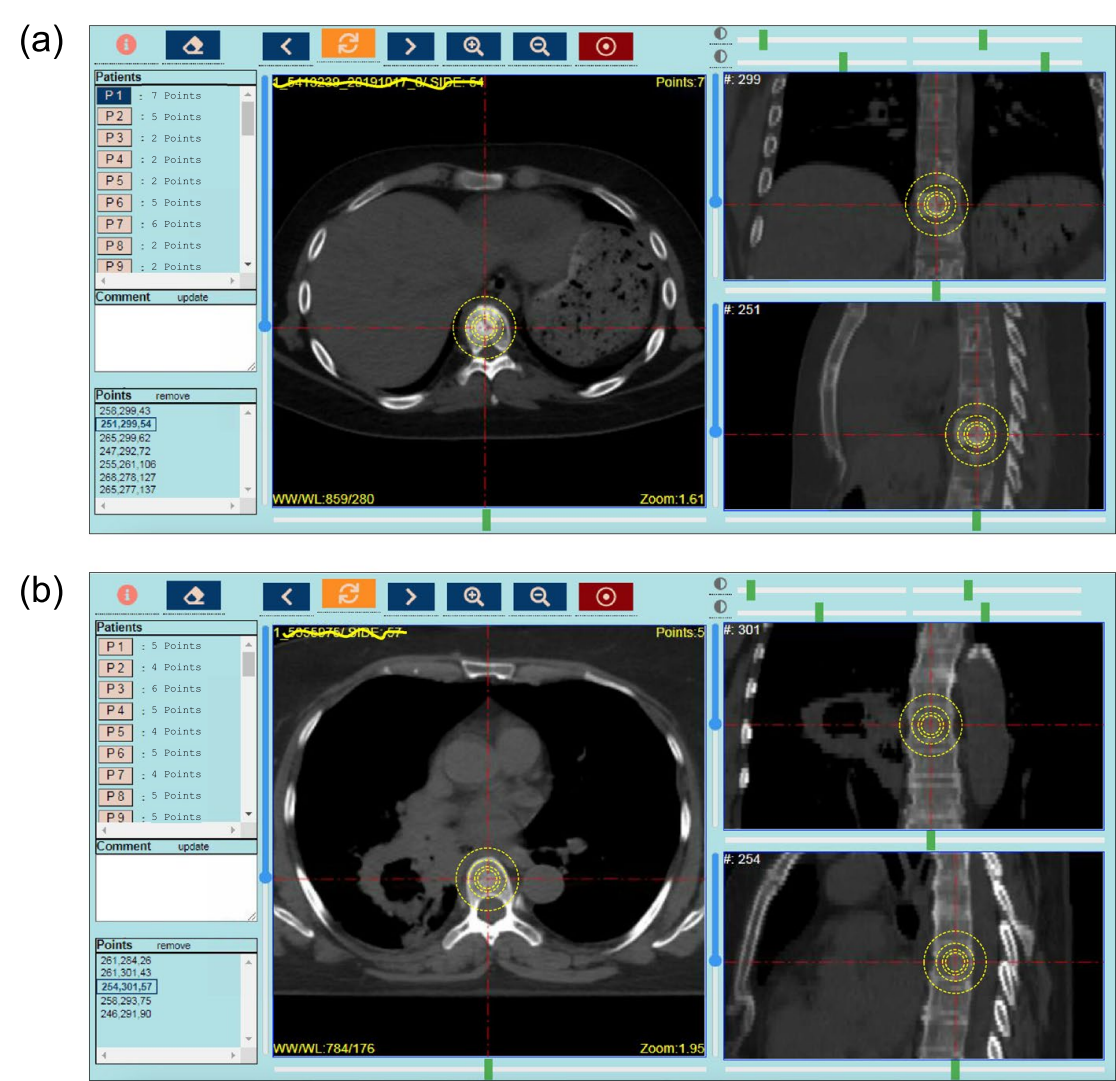
Screenshots of our diCOMBINE 3D lesion labeling web application showing expert-labeled points. (**a**) A BM lesion, and (**b**) a HB point. Cross sections of 50 mm, 30 mm, 20 mm, and 15 mm spherical ROIs are visualized with yellow dashed lines on each CT plane.

### Lesion identification

The planning-CT images of the BM patients were randomly divided into five sets using the Python |random.shuffle| module and were loaded into our custom-written 3D DICOM visualization web application (diCOMBINE^28^) for lesion identifying. diCOMBINE is an open-source software developed by our group using the Python Flask^29^ framework for DICOM 3D visualization and lesion point location labeling. The center points of BM lesions were labeled by an expert team comprising one staff radiation oncologist and four radiation oncology fellows. Each expert was asked to label BM center points in one of the five data sets, and a peer expert was tasked with reviewing them and validating the labels. A total of 631 validated BM center points were thus identified in the BM data set. Similarly, the planning-CT images of the HB patients were randomly divided into three sets and were loaded into diCOMBINE for HB labeling. One staff medical physicist and two medical physics graduate students were asked to identify HB points in one of the data sets each. When identifying HB points, the medical physicists were instructed to avoid non-metastatic skeletal complications (such as surgically-treated bone lesions). An average of four HB points were identified in each planning-CT image. Then, we asked each physicist to independently review and confirm the HB points that one of their peers had labeled. A total of 674 validated HB points were identified in this way. Screenshots of our diCOMBINE 3D lesion labeling web application are presented in Fig. 2. These BM and HB points were used as center points for our automated ROI delineation.

### Delineation of regions of interest

ROIs were automatically delineated in the planning-CT images using geometric shapes centered on the expert-identified point locations. We used four spherical (SP) and five cylindrical along the z-axis (CY) ROIs of various sizes. The characteristics of the ROIs used are specified in Table 3. The size ranges were defined to extend from the size of a large bone lesion ($\sim$15 mm)^30^ to the maximum size of a spinal vertebra ($\sim$ 50 mm)^31,32^.

**Table 3:**
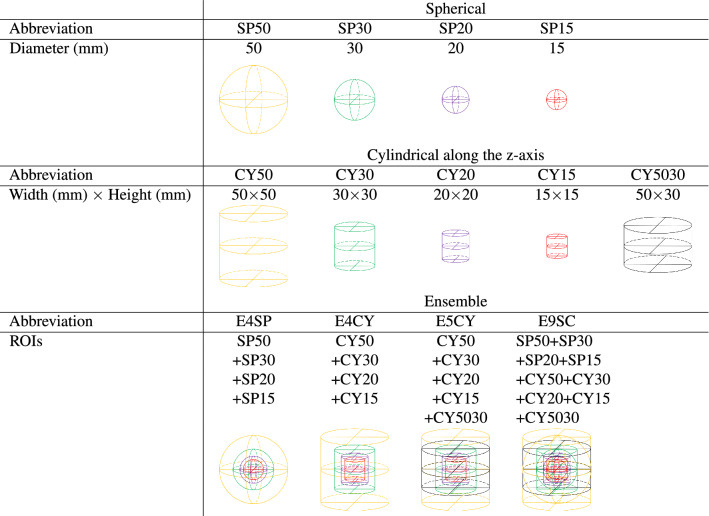
The characteristics of the ROIs used in this study.

ROIs from the planning-CT images were segmented using cylindrical and spherical ROIs with various sizes around the expert-labeled BM and HB points.

**Figure 3:**
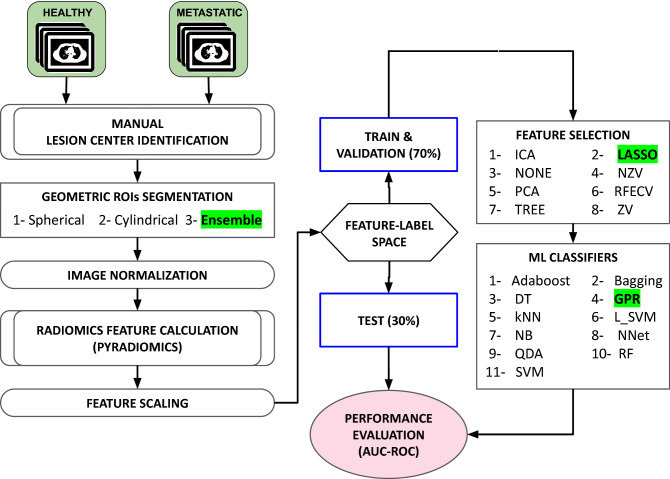
The exploration workflow for developing our radiomics-based ML models for classifying metastatic (BM) and healthy (HB) spinal bones. The best performing pipeline, as described in “Results”, is highlighted in green.

### Radiomics feature extraction

The pydicom package (https://pydicom.github.io/pydicom/stable/) was used to read DICOM CT images and normalize pixel data to Hounsfield Units. Then, the normalized CT slices were stored as 3D raster data using the pynrrd package (version 0.4.2) (https://pypi.org/project/pynrrd/0.4.2/). The pynrrd package was also used to generate 3D binary masks from each of the nine ROIs listed in Table 3. Finally, The open-source PyRadiomics package (version 3.0.1)^33^ was used to calculate the 3D quantitative radiomics features. For each of the nine ROIs listed in Table 3, we extracted 107 radiomics features from each of the planning-CT images. We did not apply any filters prior to feature extraction. These 107 features include 18 First Order, 14 Shape, 24 Gray Level Co-occurrence Matrix (GLCM), 16 Gray Level Size Zone Matrix (GLSZM), 16 Gray Level Run Length Matrix (GLRLM), 14 Gray Level Dependence Matrix (GLDM), and five Neighbouring Gray Tone Difference Matrix (NGTDM) features^34,35^. We also aggregated radiomics features from multiple ROIs to define four ensemble ROIs, including; (1) E4SP: 428 features extracted from all four spherical ROIs, (2) E4CY: 428 features extracted from the first four cylindrical ROIs, (3) E5CY: 535 features extracted from all five cylindrical ROIs, and (4) E9SC: 963 features extracted from all nine ROIs combined. Our rationale for this approach was that by aggregating features extracted from ROIs with various sizes around the BM centers, we could extract sufficient information about the BMs’ shape, size, and other characteristics and distinguish them from HBs using ML classifiers. Similar feature aggregation approaches were used in other studies^36,37^.

### Radiomics workflow

Our complete radiomics-based ML workflow is presented in Fig. 3. After extracting radiomics features for each ROI, we scaled the feature space using z-score normalization^38^. Then, we randomly divided the data set into 70% and 30% stratified training and testing sets, respectively. Each stratified set contained approximately the same BM/HB samples ratio as the initial data set. The training set was used for FS, and ML model development using 5-fold cross-validation^39^. The test set was used for the final performance evaluation. In the present study, we examined the performance of 13 FS methods and 12 ML classifiers as shown in Fig. 3 and described in the following sections.

### Feature selection methods

Radiomics calculates hundreds of features from images and some of them are redundant or are not useful for detecting BM^40^. To identify the most useful radiomics features for differentiating BM and HB, we investigated several supervised and unsupervised FS methods, including principal component analysis^41^ (PCA), fast independent component analysis^42^ (Fast ICA), zero variance threshold^43^ (VT$\_$0), near-zero variance threshold^43^, least absolute shrinkage and selection operator^44^ (LASSO) logistic regression algorithm, recursive feature elimination with cross-validation^45^ (RFECV), and decision-tree-based feature selection^46^ (TREE). For the PCA, motivated by Zack et al.^9^ we used 20, 24, and 30 features. For the LASSO method, we examined least-squares penalty ($\alpha$) values of 0.1, 0.5 and 1.0. $\alpha$ controls the stability of the selected features. A LASSO method with a larger $\alpha$ keeps fewer features (the most stable ones)^44^. For near-zero variance, we selected the variance threshold of 0.8 (VT$\_$0.8) as used by Zack et al.^9^. FS techniques were implemented using Python scikit-learn^47^ (version 0.24.2) feature selection module (https://scikit-learn.org/stable/modules/feature_selection.html). The performance of these FS methods, along with no FS, was then evaluated using 12 supervised ML classifiers.

### Machine learning classifiers

The Python scikit-learn ML package (version 0.20.4)^48^ was used to implement our ML classifiers. We used 12 supervised classification models, including the linear support vector machine^49^ (L-SVM), SVM with Radial-basis function kernel^49^ (SVM), Gaussian Naive Bayes^50^ (NB), K-Nearest Neighbors^51^ (kNN), Quadratic Discriminant Analysis^52^ (QDA), Gaussian Process Regression^53^ (GPR), Decision Tree^54^ (DT), Random Forest^55^ (RF), Bagging^55^, AdaBoost^55^, Neural network with stochastic gradient-based solver^56,57^ (NNet) and NNet with Limited-memory Broyden-Fletcher-Goldfarb-Shanno solver^58^ (NNet-LBFGS). For both NNet classifiers, we used the rectified linear unit activation function^59^ (RELU).

### Performance evaluation

The performance of our radiomics-based ML models were measured using the test data set. The standard error of calculations was reported using 5-fold cross-validation on the training data set. We used the area under the receiver operating characteristic curve^60^ (AUC) for performance evaluation. Also, we reported precision and recall for our best-performing pipeline. Matplotlib (version 3.4.3)^61^ was used to generate figures.

### Ethics declarations

This retrospective study was approved by the Research Ethics Board of the McGill University Health Centre, Montreal, Quebec, Canada, with the waiver of informed consent. We confirmed that all research were performed in accordance with the relevant guidelines and regulations.

## Results

### Radiomics feature space

**Figure 4:**
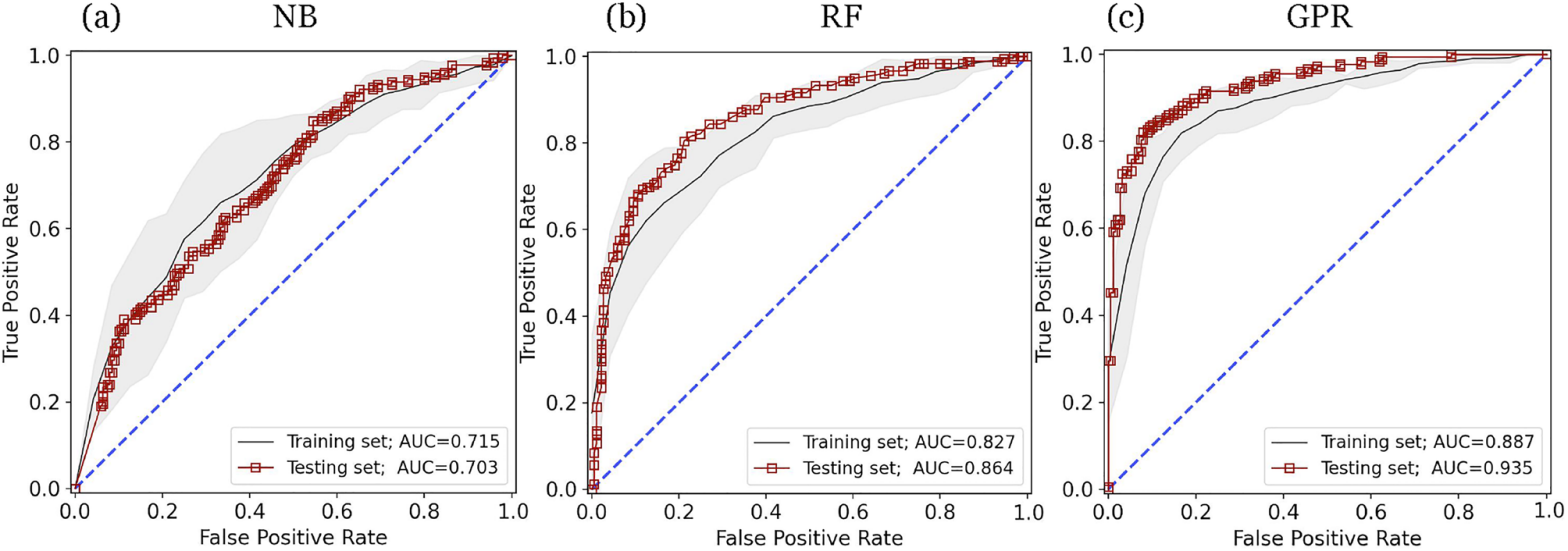
Example ROC curves for our radiomics-based ML models with the (**a**) NB, (**b**) RF, and (**c**) GPR ML classifiers Example ROC curves for our radiomics-based ML models with the (**a**) NB, (**b**) RF, and (**c**) GPR ML classifiers on the training set (black lines) and on the test set (red squares). The gray range represents the mean ROC ± SD of the 5-fold cross-validation used on the training set. Matplotlib (version 3.4.3) (https://pypi.org/project/matplotlib/3.4.3/) is used for visualizing the data. We used 20 mm spherical ROI (SP20) with no FS for this example. AUC values are presented in the legends. The 20 mm SP ROI was used for visualization purposes. Full data is available in the supplementary dataset^62^.

A JSON file of the metadata of extracted radiomics features is available in the supplementary dataset in our public repository^62^. The predictive performance of the different FS methods and ML classifiers was evaluated for each ROI on the test set using the AUC, precision, recall, and F-1 scores. Examples of receiver operating characteristic (ROC) curves are presented in Fig. 4 for the a) NB (a poor performance), b) RF (a good performance), and c) GPR (the best performance) ML classifiers on the test data set (red squares) and 5-fold validation set (pink lines) using 20 mm spherical ROI (SP20) with no FS. Note that 20 mm SP ROI was selected for visualization purposes throughout this paper for no particular reason. The effect of using the various geometric ROIs will be presented later in this paper. Raw data values, including confusion matrices, ROC graphs, and performance tables (precision, recall, F-1, ROC-AUC values on training, validation, and test sets) for all ML classifiers on all ROIs are provided in the output data folder in our public repository^62^.

**Figure 5:**
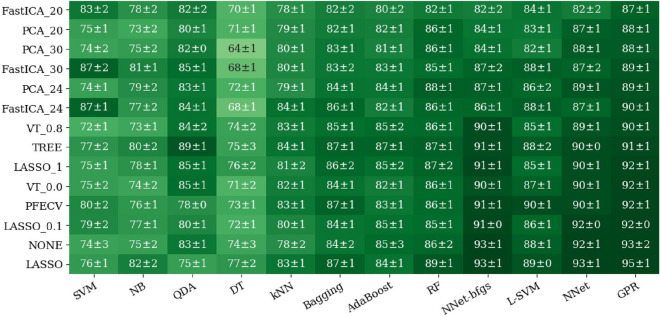
The AUC grid for different ML (x-axis) classifiers and FS methods (y-axis) combinations. The number in front of each PCA or FastICA method is the number of selected features used. The number in front of each LASSO method corresponds to the $\alpha$ penalty value (the default value is 0.5). The number in front of each VT method is its variance threshold value. Matplotlib (version 3.4.3) (https://pypi.org/project/matplotlib/3.4.3/) is used for visualizing the data.

### Effect of feature selection

An example of an AUC grid for different combinations of ML classifiers and FS methods is presented in Fig. 5 for the 20 mm SP ROI. As can be seen in Fig. 5, the best results were achieved by the GPR and NNet classifiers with LASSO FS methods. The RFECV, VT, LASSO, and TREE FS methods outperformed PCA and ICA FS methods. Overall, FS did not have much effect on the performance of the models for the 20 mm ROI. For example, for the GPR ML classifier, the performance of our model increased only 2% (from 93 to 95%) with the LASSO method compared to with no FS (NONE).

**Figure 6:**
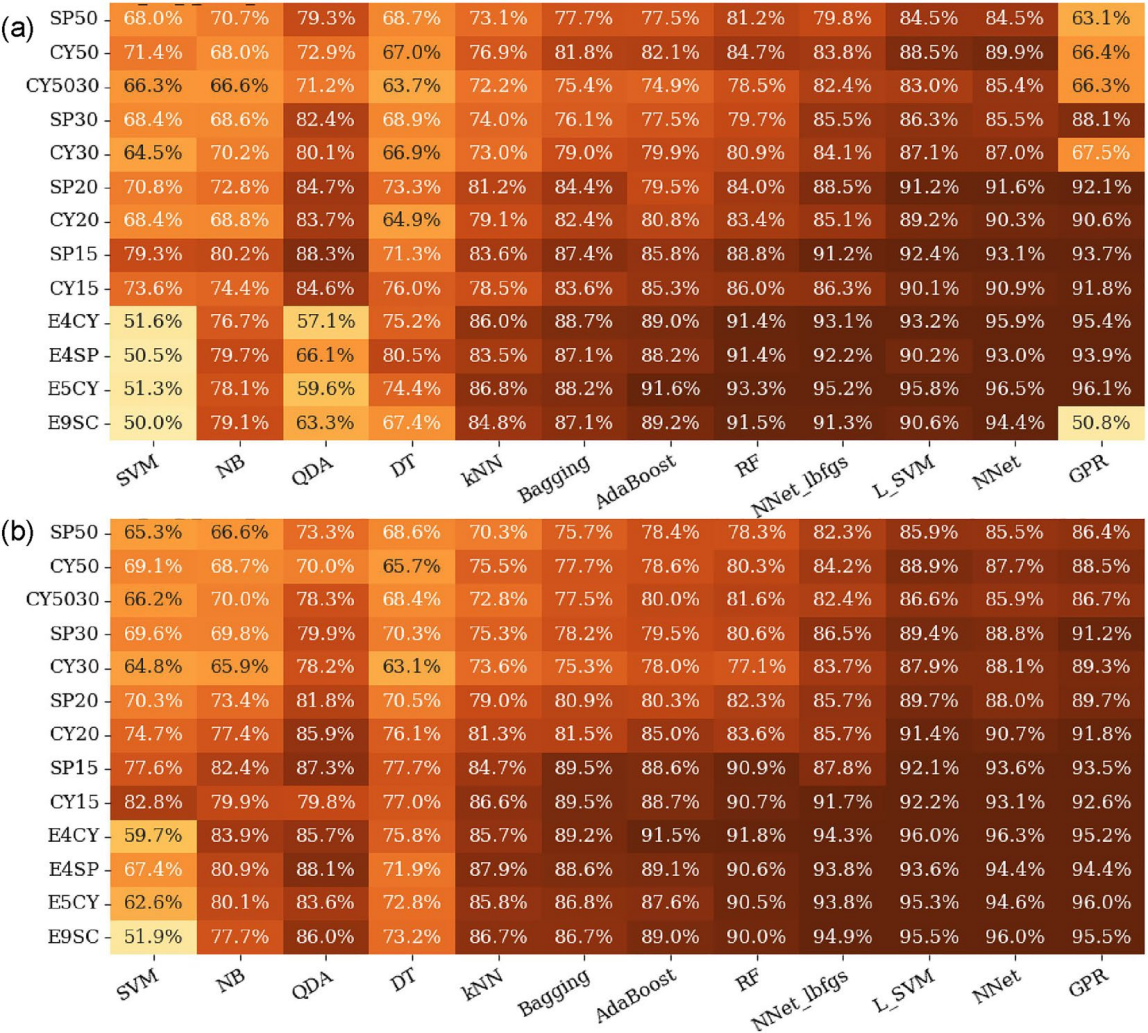
The AUC grid for different combinations of ML classifiers (x-axis) and geometric ROIs (y-axis) with various sizes and shapes (**a**) with no FS method and (**b**) with LASSO as the FS method.

### Effect of geometric ROIs

Two examples of the effect of using geometric ROIs with different sizes and shapes are presented in Fig. 6. For plot (a), we used no FS. For plot (b), we used the best performing FS method (LASSO). As can be seen in Fig. 6, the size of the ROI had a significant effect on the performance of our radiomics-based ML models. In general, a smaller ROI resulted in superior performance of models. For example, for the GPR classifier with no FS (the rightmost column of Fig. 6a), the AUC was improved from 86 to 94% when we moved from the SP50 to the SP15 ROI. SP15 resulted in the best overall performance when no FS was used. When we employed FS methods, the ensemble ROIs (like E4SP and E9SC) out-performed the single-size ROIs. This was most pronounced for the LASSO method, which is presented in Fig. 6b. The AUC grids for other FS methods are provided in the output data folder in our public repository^62^.

Comparing Fig. 6a and 6b revealed that some ML classifiers (like SVM or GPR) were more sensitive to the use of FS than others (like NNet or RF). Also, we noticed that FS was more important when using large ROIs (such as SP50 or CY50) or ensemble ROIs (such as E4SP or E9SC).

**Figure 7:**
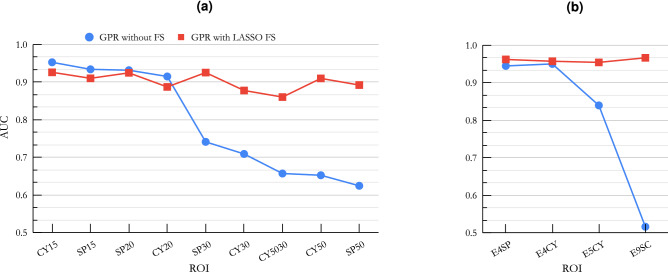
An example of AUCs versus the volume of the ROI for (**a**) single geometric ROIs and (**b**) for ensemble ROIs (Ref. Table 3). For this graph, we used our best performing ML classifier (GPR), with our best performing FS method (LASSO), and without FS method.

To visualize the effect of the size of ROI on the performance of our models, in Fig. 7 we show the AUCs of our best performing ML classifier (GPR) for (a) single geometric ROIs (sorted by volume), and (b) for ensemble ROIs (sorted by total volume). To show the effect of the use of FS, we plotted the results without FS (blue circles), and with our best-performing FS method (LASSO) (red squares). It can be seen that a smaller ROI resulted in a better performance. Also, FS was more important for larger ROIs (like SP50 and CY50) and ensemble ROIs (like E9SC).

**Figure 8:**
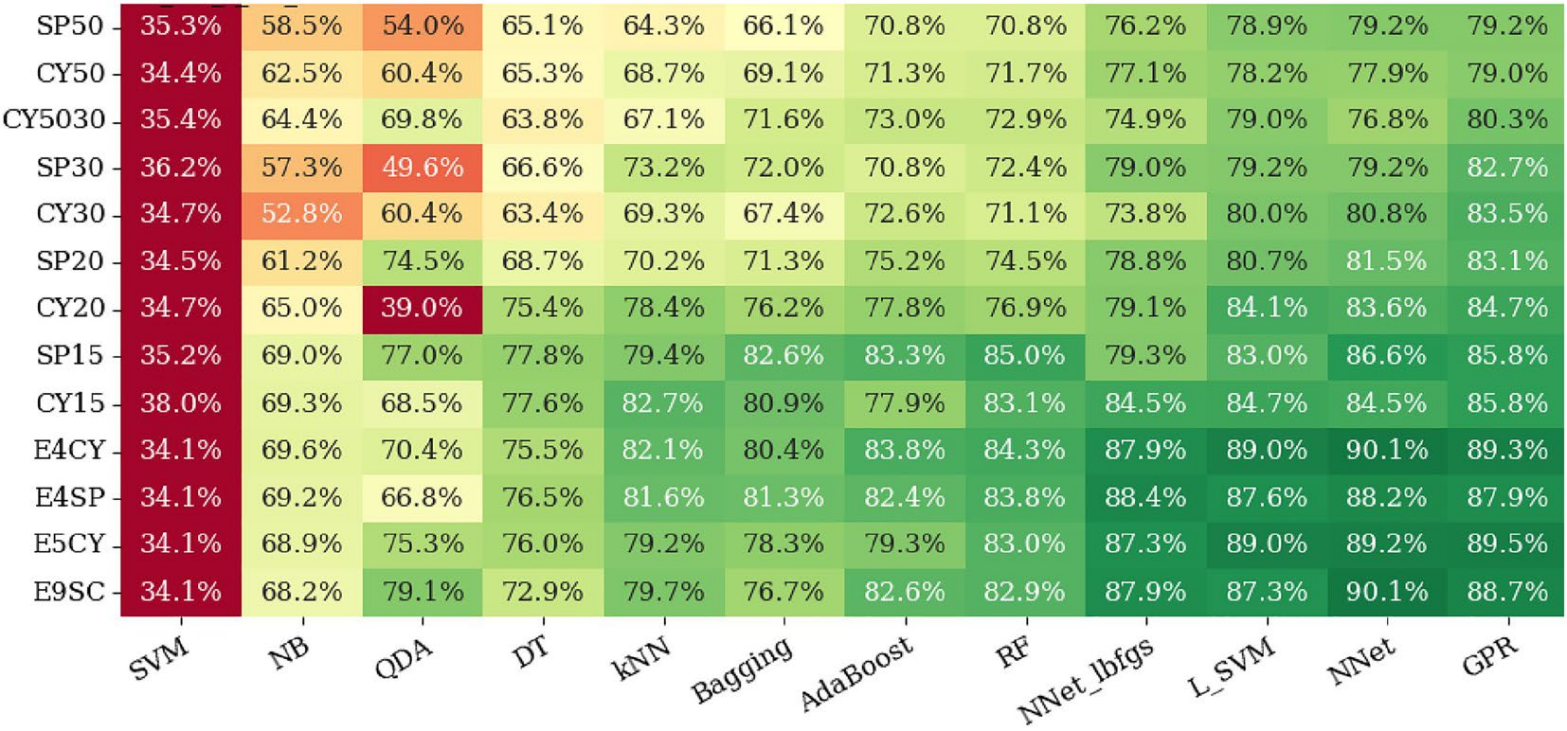
The F-1 score grid for different combinations of ML classifiers (x-axis) and ROIs (y-axis) with different sizes and shapes with LASSO FS method.

The grid of the F-1 scores for the best performing FS method (LASSO) is presented if Fig. 8. The GPR, NNet, and L-SVM classifiers achieved 0.9 F-1 score in detecting BM using the ensemble ROIs. The AUC, precision, recall, and F1 score of our best performing pipeline, corresponding to the E9SC ROI, LASSO FS method, and GPR ML classifier, were 96%, 92%, 91%, and 0.9, respectively. The performance of our models for all combinations of FS methods, ML classifiers, and ROIs are provided in the supplementary dataset^62^.

## Discussion

In this study, we investigated the feasibility of using a single-point-based geometric ROI to develop a radiomics pipeline to distinguish BM and HB locations in planning-CT images of cancer patients with BM. We investigated various FS methods, and ML classifiers using point-based geometric ROIs with various shapes and sizes.

The time and effort needed for manual 3D segmentation of ROI are significant limitations to achieving large real-world image data sets. This, in turn, hinders the generation of generalizable radiomics-based prognostic ML models^63^ for use in the clinic. Another limitation of manual lesion segmentation is inter-observer variability, which has been shown to have a significant impact on the performance and reproducibility of radiomics-based pipelines^64^. Furthermore, manual segmentation tools, designed for radiation therapy treatment planning, intend to load one patient at a time. Therefore, switching between patients is another time-consuming process that slows down the lesion delineation for multiple patients in the research context^65^.

Our in-house-developed open-source 3D DICOM visualization and lesion identification tool (diCOMBINE^28^) allowed our collaborating radiation oncologists to quickly review planning-CT images of several hundred patients and efficiently identify 676 BM centers. They found diCOMBINE fast and easy to use, allowing each expert to label around 150 lesions per hour. Based on our experts’ anecdotal experience, single-point-based geometric ROI delineation was 10–15 times faster than full manual 3D segmentation. These lesion centers were used to generate ROIs automatically. Defining point-based geometric ROIs, instead of full 3D manual segmentation of the ROIs, allowed us to rapidly generate a large sample set, minimize expert imposed uncertainties, and investigate the effect of the size and shape of the ROIs in the performance of our radiomics models. Besides, our point-based radiomics models will allow us to study the feasibility of building an automated BM-identifying pipeline. To the best of our knowledge, no studies on automated BM delineation have been published previously.

Radiomics extracts hundreds of features from an ROI. However, these features are generally highly correlated and contain much noise. Therefore, it is essential to apply proper FS methods to achieve a robust radiomics-based ML pipeline. Among the seven FS methods we examined in this study, we found that PCA and ICA resulted in lower AUC values than the VT, LASSO, and TREE FS methods. One reason for this difference was that VT, LASSO, and TREE methods automatically defined the optimal number of features, while in PCA and ICA, the number of features was predefined. For highly-correlated features, the optimal number of features (f) is roughly proportional to the square root of the sample size (n)^66^. Accordingly, 30 features would appear to be less than the optimal number of features for our sample size ($f=\sqrt{n}=\sqrt{1305}=36$). For studies with small sample sizes, such as Zhang et al.^9^, that used 112 samples, PCA with 10 features seems to be a suitable FS method. We also noticed that the effect of the FS method depends on the selected ML classifier. For ML classifiers that had built-in FS methods (i.e., RF and NNet), applying FS methods in some cases worsened the overall performance of the model. Inversely, for ML classifiers that did not have built-in FS methods (i.e., GPR), adding FS had a significant effect on the performance of the ML classifier. The effect of the FS method was more significant when working with ensemble ROIs that had many more features. For example, the AUC value for the GPR ML classifier using the E9SC ROI (963 features) improved from 0.52 to 0.97 when the LASSO FS method was used, as shown in Fig. 6.

Among the ML methods we examined in this study, we found that GPR, NNet, SVM, and RF resulted in the highest AUC values and F-1 scores. We showed that the GPR classifier outperformed the NNet classifier for most ROIs. However, for the ensemble ROIs (in which the number of features was large), GPR required a proper FS method (i.e., LASSO). The dimensionality issue of GPR classifiers and their requirement for FS was also discussed in the literature^67,68^.

We found that our radiomics-based ML models performed slightly better on spherical ROIs compared to cylindrical ROIs of similar volumes. More significantly, we found that the smaller ROIs (15 and 20 mm) resulted in better performance compared to the larger ROIs (30 and 50 mm) (Fig. 6). This might be due to the fact that in larger ROIs there are probably more outlier features captured from bone or organs/tissue surrounding the lesion of interest. Performances of our models did not improve considerably by decreasing the size of ROI below 20 mm, which is roughly the size of a large BM lesion^30^. As can be seen in Fig. 7, our models performed better on the ensemble ROIs compared to the single ROI when used with FS methods. This could be due to having many features in the ensemble ROIs. For example, the E9SC ROI contains 9$\times$107 = 963 features. For such a prominent feature space, FS methods become very important.

Although various radiomics pipelines have been previously developed and reported to classify bone lesions, our radiomics-based ML pipeline, reported here, offers several advantages compared to preceding efforts, mainly in the context of palliative radiotherapy planning. First, we, pragmatically, used planning-CT images of cancer patients for extracting radiomics features, whereas prior studies used hybrid modalities or diagnostic-CT images (as listed in Table 1). Hybrid modalities allow the development of high-quality prognostic pipelines. However, these pipelines are less clinically applicable in palliative radiotherapy treatment planning for BM, which is often primarily based on a patient’s planning-CT scan. Second, all ML classifiers presented in the prior studies were restricted to full 3D segmentation of the lesion volumes. In the real-world clinical workflow for palliative radiotherapy of BM, it is common to use single-slice or lesion-center-based treatment planning with radiation oncologists often defining treatment field limits rather than lesion contours. Therefore, pipelines that require full 3D segmentation of ROI have limited application in real-world palliative radiotherapy^65^. Moreover, 3D segmentation of the ROI is a time-consuming bottleneck that likely compelled all the prior studies to train and test their radiomics pipelines with limited sample sizes. Training on a small sample size diminishes the generalizability and clinical applicability of a radiomics pipeline. In comparison, our point-based pipeline allowed us to avoid the labor-intensive manual segmentation step and train and test our pipeline on a large data set. Finally, in this study, we investigated the effects of FS methods, and ML classifiers in achieving the optimal prognostic model using geometric ROIs. To the best of our knowledge, no prior study performed such a comprehensive optimization.

Our study had some limitations. First, we selected BM and HB from two sets of separate patients. This selection might drive the risk of potential susceptibility to bias if there is a systematic difference between the two sets of images. However, our rationale for using non-metastatic cancer patients to select HBs was to eliminate the possibility of error in labeling HBs by our medical physicists. Second, our collaborating medical physicists could not identify non-metastatic skeletal complications from metastatic bone lesions. Therefore, the non-metastatic skeletal complications (i.e., surgically-removed lesions or bone islands) were ignored when labeling HB points. A solution for this problem would be using pathology data to identify non-metastatic and metastatic lesions but this would significantly increase the required effort. Third, we used a nearly balanced data set of HB and BM patients in this study. However, having an imbalanced sample ratio is common in many real-world radiation oncology outcome data sets^69,70^. A study with an imbalanced data set is required to evaluate the effect of sample imbalance when building high-performance real-world radiomics-based ML models^71–73^. Forth, while using geometric ROIs significantly simplified the lesion delineation procedure, it ignored some lesion details such as size and shape. One alternative that can be explored as future work is to use deep-learning-based ROI segmentation. Finally, we used single-center planning-CT images from 359 patients in this retrospective study. A multi-center study with a more extensive data set is required to test the generalizability of our radiomics pipeline. Such a big data set would allow us to try more robust deep learning ML classifiers^74,75^ to build an AI tool to scan patients’ planning-CT images and identify BM lesions automatically. The present work provides strong motivation to pursue such a multi-center study.

## Conclusion

We demonstrated that our radiomics-based ML models can successfully distinguish between metastatic and healthy bones in planning-CT images using lesion-center-based geometric ROIs. Our results suggest that the GPR classifier with ensemble ROIs is particularly promising for the differentiation of BM and HB. Optimum pipeline performance was obtained using elimination-based FS methods such as LASSO. Our results demonstrate that radiomics features obtained from a lesion-center-based geometrical ROI may be sufficient to train radiomics-based ML classifiers to distinguish between bone lesions when full 3D segmented ROIs are not available. This opens the door to big data artificial intelligence research for cancer patients with BM.

## Data Availability

The supporting dataset is provided as a figshare repository^62^. This repository contains three files: (1) “featurespace_metadata.json.zip” file that includes radiomics features extracted from 1273 spinal lesions (healthy or metastatic) from radiotherapy planning-ct images using geometrical regions of interest (ROIs). (2) “output.zip” folder that contains the results of our radiomics-based machine learning models that were validated and tested using several FS, and ML on single-point-based geometric ROIs with various shapes and sizes. (3) A README.md file that is provided to explain the information about the data structure and file naming patterns.
